# Creatine kinase elevation and discontinuation of clozapine: fear-driven clinical practice in a forensic case with treatment-resistant schizophrenia and persistent violent behaviour

**DOI:** 10.1192/bjo.2020.134

**Published:** 2020-12-04

**Authors:** Hein Bokern, Niels Jonker, Dan Cohen

**Affiliations:** Dr. S. van Mesdag Forensic Psychiatric Center, Groningen, The Netherlands; Certe, Wilhelmina Hospital, Assen, The Netherlands; Community Psychiatry Heerhugowaard, Mental Health Organization North-Holland North, The Netherlands

**Keywords:** Schizophrenia, clozapine, side-effects, creatine kinase, neuroleptic malignant syndrome

## Abstract

In forensic settings, the most common indication for clozapine is treatment-resistant schizophrenia (TRS). Clozapine has also been shown to be effective in reducing hostility, aggression and violence in patients with schizophrenia and is of benefit in comorbid substance use disorders. The decision to initiate or to discontinue recently initiated clozapine can have a profound beneficial or detrimental influence on the lives and safety of patients and the staff caring for them. We present a case in which treatment with clozapine proved effective in spite of earlier repeated discontinuation of clozapine out of fear of neuroleptic malignant syndrome (NMS). Elevation of creatine kinase was deemed to be indicative of NMS in the absence of clinical signs of NMS. In somatic medicine, it is well known that creatine kinase elevation has many causes, most of them non-harmful. Collaboration with clinical chemistry was shown to be very useful, if not essential; research in the 1980s found replicated evidence for both sex and race differences in creatine kinase levels. In addition, substantial intra-individual variation has been found over time in healthy individuals. The creatine kinase levels of this patient of African descent were within normal limits for the African population. Baseline creatine kinase assessment and repetition of this assessment after 2 weeks with careful interpretation are recommended in all clozapine-treated patients. The authors advocate the introduction of evidence-based creatine kinase cut-off points that reflect the biological differences between the sexes and among races. More intensive contact between psychiatrists and clinical chemist can facilitate faster diagnosis and better treatment.

## Case Presentation

*Mr T, a 37-year-old man of African origin with a 7-year history of forensic hospital admissions for theft, verbal threat and assault from the age of 16, was diagnosed with a psychotic disorder and an aggressive behaviour disorder. Two antipsychotics proved ineffective. Clozapine was twice initiated and discontinued: a steep rise in creatine kinase (plasma levels unavailable) raised concerns about the development of neuroleptic malignant syndrome (NMS). During a subsequent hospital stay in a general mental hospital, the diagnosis of psychosis was dropped and an antisocial personality disorder was diagnosed instead. As he reoffended soon after discharge, with threatening behaviour and aggression with a weapon, he was involuntarily admitted to a high-security forensic psychiatric centre (FPC). Addition of 300 mg clozapine to 30 mg olanzapine daily proved ineffective for his unpredictable aggressive behaviour, resulting in prolonged seclusion. Clozapine plasma levels remained below the lower threshold of 350 μg/L, possibly owing to non-adherence. Switching from oral to monthly injected long-acting medication (100 mg haloperidol and 405 mg olanzapine) failed to improve his condition. After committing serious physical violence, Mr T was sent to the high intensive care unit of our FPC. On admission, he reported hearing malevolent voices and showed paranoid, hostile and irritable behaviour. Two psychiatric nurses and two well-trained security officers looked after him outside his room. The presence of two therapy-resistant disorders, psychosis and aggression, argued strongly in favour of a fourth clozapine treatment. Use of valproate or lithium was not considered. We convinced him that his ‘allergy to clozapine’ was surmountable, and clozapine was reinitiated. Baseline creatine kinase was 203 U/L (normal range <200 U/L). The long-acting injections were stopped and replaced with 5 mg oral haloperidol daily. Adjunct clorazepate, 50 mg daily, was continued. After several weeks, Mr T refused clozapine treatment and blood sampling. We obtained permission for involuntary drug treatment, whereupon he changed his mind and agreed to continue with oral clozapine and blood sampling. Close observation of daily intake of 900 mg clozapine resulted in therapeutic plasma levels (350–450 μg/L) and remarkable changes: after 5 weeks, his positive psychotic symptoms – irritability, hostility and disregard for the rights or safety of others – substantially diminished. Clorazepate was discontinued. After 6 months, negative psychotic symptoms dominated the clinical picture; he was transferred to a medium-security ward without security officers. Without intensive exercise or sporting activity in the days before blood sampling, his creatine kinase level had risen to 451 U/L. Although he felt tired and sleepy, he felt no muscle pain and had normal body temperature. He was not disorientated but alert and at ease. In the absence of clinical signs of NMS, the creatine kinase elevation was interpreted as benign and clozapine was continued. After a week, his creatine kinase level decreased to 382 U/L*.

*In the four consecutive years of clozapine treatment, he remained stable without aggressive incidents, even during unguided leave outside the hospital*.

## Discussion

The prescription rate for clozapine is 50% in our FPC. Treatment-resistant schizophrenia (TRS), for which clozapine is the standard recommended treatment, is the most common indication. Clozapine has also been shown to be effective in reducing hostility, aggression and violence in patients with schizophrenia and is of benefit in comorbid substance use disorders.

Psychotic forensic patients often lack insight in their disease and tend not to adhere to antipsychotics. As forensic patients often forcefully resist clozapine treatment and threaten legal or disciplinary action, psychiatrists are more apprehensive about clozapine's potentially lethal side-effects. Development of fever and tachycardia combined with fatigue, muscular pain and weakness in the initial phase of treatment should alert the psychiatrist. Clinical guidelines, such as the Dutch clozapine guideline,^1^ provide clinicians with essential information about leukocyte monitoring and the treatment of common (hypersalivation, constipation) and uncommon (myoclonus, hypotension) side-effects of clozapine. When leukopenia has been excluded, myocarditis, NMS and rhabdomyolysis must be considered. Clozapine-associated NMS is a rare condition but must be identified when present.

The enzyme creatine kinase facilitates the transfer of a phosphoryl group from phosphocreatine to ADP, thereby transforming ADP into ATP. This process results in the local production of ATP for the most energy-demanding processes in the human body, such as muscle movement. In healthy adults, plasma creatine kinase consists of three subfractions, the dimers CK-MM, CK-MB, and CK-BB: CK-MM originates in muscle tissue, CK-MB in the myocard and CK-BB in the brain. As CK-MM makes up >97% of total creatine kinase, total creatine kinase in plasma or serum is commonly used for diagnosis of non-cardiac pathology. The activity of creatine kinase is measured by a standard biochemical laboratory test that can be ordered 24/7 in most clinical chemistry laboratories.

In our patient, a sharp increase in creatine kinase levels led to clozapine discontinuation twice. This laboratory finding was wrongly equated with muscle damage, which in turn was interpreted as a foreboding of NMS. The consensus diagnostic criteria for NMS^2^ are recent dopamine antagonist exposure or dopamine agonist withdrawal, hyperthermia, rigidity, sympathetic nervous system instability, tachycardia with tachypnoea, and raised creatine kinase levels (four times the upper limit). Historically, raised creatine kinase levels were considered a major manifestation of, if not the essential test for, NMS.^3^ Creatine kinase levels higher than 1000 IU/L were, in the absence of intramuscularly administered medication, considered suspect for the syndrome or associated organic pathology.^4^ However, strongly elevated creatine kinase levels (>1000 IU/L in three cases) were measured in 70% of patients (*n* = 10) who developed fever on psychotropics without NMS.^5^ Clozapine initiation caused creatine kinase elevation without any clinical features of NMS.^6^

In a study comparing patients who did or did not develop NMS, creatine kinase levels were raised in all patients (*n* = 10) with the syndrome, 60% of whom had levels higher than 1000 IU/L.^7^ However, creatine kinase levels were also raised in 67% of patients (*n* = 30) without the syndrome (20% >1000 U/L); this was associated with intramuscular injections and/or physical restraint. In a separate study (*n* = 17), creatine kinase levels that became raised after intramuscular injections returned to normal within 72 h after the last injection.^8^

In short, raised creatine kinase levels in the absence of other clinical signs and symptoms tend to be a non-specific finding and do not constitute NMS. Indeed, a patient with fully developed NMS and normal creatine kinase levels has been reported,^9^ as has a patient with atypical NMS without fever but with raised creatine kinase levels.^10^ Not only do the clinical characteristics of NMS with clozapine and other antipsychotics not differ,^11^ but clozapine has been given successfully to eight out of nine patients who developed NMS on other antipsychotics.^12^ Atypical presentation with little, no or delayed creatine kinase elevation^13^ can complicate and delay the diagnosis of NMS.^14^ Clozapine and second-generation antipsychotics (SGA) in general are said to induce more varied presentations of the syndrome. The timely and accurate diagnosis of atypical NMS can therefore be a difficult and challenging task for the clinician.

Unfortunately, knowledge about more common and benign creatine kinase elevation has shifted to the background. For instance, primary antipsychotic-induced creatine kinase elevation or massive asymptomatic creatine kinase elevation (MACKE), which is characterised by a transient increase (2–20 times the upper limit) of skeletal muscle creatine kinase without excessive exercise or trauma, was first reported in acutely psychotic patients in 1968.^15^ In 10% of patients (*n* = 121) treated with SGA, massive increases in creatine kinase were observed. They appeared to be more frequent in patients on SGA maintenance, and were higher and persisted longer than in patients treated with first-generation antipsychotics (FGA).^16^ Increases above the upper limit were present in 17% of patients on SGA (*n* = 36) versus none on FGA (*n* = 13).^17^ Selectivity is another point: in one case, creatine kinase increased with olanzapine and quetiapine but not with clozapine.^18^ Periodic monitoring of clinical symptoms and creatine kinase levels has been recommended by several studies.^18,19^ A systematic review of 42 published cases of MACKE – nine with clozapine treatment – found a prevalence of 2–7%.^20^ MACKE was more frequent in males and in SGA-treated patients. The presence of symptoms was strongly related to myoglobinuria but unrelated to the magnitude of the creatine kinase elevation. The probability of a new creatine kinase elevation was not significantly higher after rechallenge than after switching to another antipsychotic. In the case of clozapine, a rechallenge could be considered, mainly because of the lack of alternatives. Measurement of creatine kinase is advised in patients on antipsychotics who develop muscle symptoms.

Strenuous physical exercise in the previous 24 h can significantly raise creatine kinase levels, which gradually return to normal over the course of a week. The magnitude of the increase depends on the nature and duration of the exercise, and on the training status of the person (with greater increases in the untrained), and there are high inter-individual variations.^21^ Physical trauma (including epileptic fits or intramuscular injections), hypothyroidism, and rheumatic or neuromuscular diseases can increase creatine kinase levels.^22^ Statins are responsible for a 2–10-fold increase in CK levels in about 5% of treated individuals, probably as a result of low-level myopathy or rhabdomyolysis.^23^ Macro creatine kinase and idiopathic elevated serum creatine kinase are less common causes of creatine kinase elevation.^24^ Macro creatine kinase is an abnormal enzyme complex with an atypically high molecular mass (detectable by electrophoresis^25,26^) and reduced clearance, resulting in abnormally high blood levels of creatine kinase. Macro creatine kinase is present in 4% of patients with asymptomatic or minimally symptomatic elevated creatine kinase. Idiopathic elevated creatine kinase is a persistent creatine kinase elevation with negative findings on examination, testing (electromyography, nerve conduction and muscle biopsy) for a neuromuscular disease and family history for neuromuscular disease.^27^

Although more has been learned about changes in creatine kinase levels in recent years, two findings have not yet percolated down to current clinical psychiatry: the differences between the two sexes and those among races. This observation is explicitly stated in only two of the studies^16,18^ discussed above. In 1974, creatine kinase activity was shown to be higher in African Americans than in Caucasians.^28^ In 1983, Wong et al^29^ found high (52–520 U/L) creatine kinase levels in African American men; intermediate (35–345 U/L) levels in Caucasian men and African American women; and low (25–145 U/L) levels in Caucasian women. The sex and ethnic differences are clear, but these values cannot be used in clinical practice because they date from before assay standardisation and the use of international units. A second study^30^ confirmed differences in creatine kinase levels between the sexes and between people of Caucasian and African origin, while people of Hispanic origin showed creatine kinase levels in between those of the other two groups. These differences in creatine kinase concentrations were similar in tissue and plasma.^31^ The National Health and Nutrition Examination Survey showed significantly larger differences between the 90th, 95th and 97.5th percentiles in Black compared with White males (Table 1).^32^ Evidence suggests a non-Gaussian distribution of creatine kinase values in people of African origin.^32^ Although possible confounders (differences in muscle mass and renal clearance) have been excluded, a satisfactory explanation of this phenomenon is still lacking.

**Table 1:** Creatine kinase levels in the US population^32^

Group	90th percentile	95th percentile	97.5th percentile	Unit
Black male	544	712	1001	IU/L
White male	237	312	382	IU/L
Black female	254	323	487	IU/L
White female	142	188	295	IU/L

To complicate matters further, intra-individual variability in creatine kinase levels in healthy individuals can be as high as 15.4%.^33^ Correct interpretation of creatine kinase levels requires reference values in male and female persons of different ethnic origins, and repeated measurement of creatine kinase levels over the course of 2–3 weeks.

In Box 1, different causes of creatine kinase elevation are summarised. In our patient, we considered the changes in creatine kinase levels during clozapine treatment to represent normal intra-individual variations for his ethnicity and sex, although benign clozapine-induced primary creatine kinase elevation could not be ruled out.

Box 1 Causes of CK elevation

Recent physical exercise
Medication
Alcohol or drug use
Myocardial damage
Accidental or iatrogenic damage
  Trauma
  Surgery
  Intramuscular injection
Non-Caucasian ethnicity
Myopathy
  Inflammatory
  Infectious
  Metabolic
  Endocrine
Rhabdomyolysis
Neuromuscular or rheumatic disorder
Muscular dystrophy
Malignant hyperthermia
Abnormal (macro) creatine kinase
Neuroleptic malignant syndrome
Miscellaneous
  Epilepsy
  Dystonia
  Catatonia
  Motor neuron disease
  Asymptomatic elevations of creatine kinase
  Kidney disease

## Conclusion and recommendations

This case illustrates several important issues. First, clozapine was the only effective drug for two treatment-resistant disorders: schizophrenia and aggressive disorder. Second, insufficiently substantiated clozapine discontinuation can be detrimental to the patient. Third, a fixed upper level for normal serum creatine kinase ignores evidence of clinically relevant sex and ethnicity differences in creatine kinase levels, which are lowest in Caucasian women and highest in African males. In our patient, creatine kinase levels were within the normal limits for African males.

Based on our findings and experience, we have four recommendations for clinical practice.

- Recommendations concerning clinical decision rules are complicated by the large variety in ethnic backgrounds in our patient population. On the one hand, we have clear data that show that higher creatine kinase levels in the healthy African population are completely benign and do not indicate any pathology. On the other hand, we lack a state of the art and adequately powered assessment of the upper (and lower) creatine kinase limits in different ethnicities and in male and female populations. Within these limits, we advise measuring creatine kinase levels before clozapine initiation and repeating this assessment after 2 weeks of clozapine treatment, and interpreting the results carefully in the context of differences between ethnic groups and biological variations.
- Evidence-based sex- and ethnicity-specific creatine kinase cut-off points should be established.
- Clinical chemists should educate psychiatrists regarding individual variations and accurate personalised reference values. Racial differences in creatine kinase levels and common causes of high creatine kinase are established knowledge in clinical chemistry and internal medicine, but the effects on the diagnosis of NMS in psychiatry are not. In these and other cases concerning psychiatric patients, more intensive contact between psychiatrists and clinical chemists could facilitate faster diagnosis and better treatment. In general, we recommend easily accessible contact between psychiatrists and clinical chemists, as their knowledge and skills are complementary.
- Consultation should be used to improve clinical decision-making. Clozapine discontinuation based only on laboratory findings, that is, without clinical signs, is unjustified and can prolong patients’ suffering. In this case, unjustified clozapine discontinuation probably prolonged the patient's suffering for several years.

In the future, we hope and expect genetic screening to improve the currently imprecise trichotomy of Caucasian, Asian or African origin into a more sophisticated, evidence-based differentiation.

Ethical approval was not required and was not obtained for this case report. The patient has given his verbal and written consent.

## Supplementary material

For supplementary material accompanying this paper visit http://dx.doi.org/10.1192/bjo.2020.134. click here to view supplementary material
